# Supplementary material for: A Source of Systematic Errors in the Determination of Critical Micelle Concentration and Micellization Enthalpy by Graphical Methods in Isothermal Titration Calorimetry
Source: Entropy (Basel). 2021 Feb 18;23(2):236. doi: 10.3390/e23020236 (PMC7922405; doi:10.3390/e23020236)
Supplement: Supplementary file 1 [file entropy-23-00236-s001.pdf]

Table S1. Critical micelle concentration of C16-TAB taken from the bibliography using isothermal titration calorimetry with the same experimental design.

Reference	Temperature (°C)	CMC	Stock Solution Concentration in the Syringe	Method Employed to Calculate de CMC
23	30	0.93 mmol/kg	Not exactly reported (10 times the CMC)	Williams's method using the cumulated heat
33	25	0.9 mmol/kg	Not exactly reported. (more than 20 times de CMC)	Inflexion point (Phillips's method)
34	25	0.94 mM	5 mM	Inflexion point (Phillips's method)
35	25	0.888 mM	Not reported	Inflexion point (Phillips's method)
	30	0.907 mM		
	35	0.929 mM		
	40	0.977 mM		
	45	1.041 mM		
	50	1.104 mM		
36	30	1.03 mM	30.94 mM	Inflexion point (Phillips's method)
37	28	0.925 mM	Not exactly reported (10 or 15 times de CMC)	Inflexion point (Phillips's method)
	30	0.950 mM		
	35	0.975 mM		

Table S2. Micellization enthalpy of C16-TAB taken from the bibliography using isothermal titration calorimetry with the same experimental design.

Reference	Temperature (°C)	Micellization Enthalpy (kJ/mol)	Stock Solution Concentration in the Syringe	Method Employed to Calculate de CMC
23	30	−7.1	Not exactly reported (10 times the CMC)	Difference evaluated at the CMC between the extrapolations of micellar region and monomer region
28	25	−10.5	Not reported	Difference evaluated at the CMC between the extrapolations of micellar region and monomer region
	30	−11.7		
	35	−14.1		
	40	−17.5		
	45	−21.4		
33	25	−8.1	Not exactly reported (20 times the CMC)	Difference evaluated at the CMC between the extrapolations of micellar region and monomer region
34	25	−6.9	5 mM	Difference evaluated at the CMC between the extrapolations of micellar region and monomer region
35	25	−7.894	Not reported	Difference evaluated at the CMC between the extrapolations of micellar region and monomer region
	30	−11.08		
	35	−14.00		
	40	−16.78		
	45	−19.67		
	50	−22.15		
36	30	−13.90	30.94 mM	Difference evaluated at the CMC between the extrapolations of micellar region and monomer region
37	28	−9.40	Not exactly reported (10 or 15 times de CMC)	Difference evaluated at the CMC between the extrapolations of micellar region and monomer region
	30	−11.12		
	35	−14.41		
39	25.0	−12.0	565 mM	Direct subtraction of the momomer region from the micellar region
	29.9	−13.6		
	40.0	−18.5		
	50.1	−23.8		
